# Oral health literacy, knowledge and perceptions in a socially and culturally diverse population: a mixed methods study

**DOI:** 10.1186/s12889-023-16381-5

**Published:** 2023-07-28

**Authors:** Shalinie King, Ayesha Thaliph, Liliana Laranjo, Ben J. Smith, Joerg Eberhard

**Affiliations:** 1 Westmead Applied Research Centre and the Sydney Dental School, Faculty of Medicine and Health, The University of Sydney, Sydney, Australia; 2 Westmead Applied Research Centre, Faculty of Medicine and Health, The University of Sydney, Sydney, Australia; 3 Sydney School of Public Health, and the Westmead Applied Research Centre, Faculty of Medicine and Health, The University of Sydney, Sydney, Australia; 4 Sydney Dental School and the Charles Perkins Centre, Faculty of Medicine and Health, The University of Sydney, Sydney, Australia

**Keywords:** Oral health literacy, Oral health knowledge, Oral health disparities, Held-14, Poor oral health, Oral health

## Abstract

**Background:**

Poor oral health literacy has been proposed as a causal factor in disparities in oral health outcomes. This study aims to investigate oral health literacy (OHL) in a socially and culturally diverse population of Australian adults visiting a public dental clinic in Western Sydney.

**Methods:**

A mixed methods study where oral health literacy was assessed using the Health Literacy in Dentistry scale (HeLD-14) questionnaire and semi-structured interviews explored oral health related knowledge, perceptions and attitudes. Interviews were analysed using a thematic approach.

**Results:**

A sample of 48 participants attending a public dental clinic in Western Sydney was recruited, with a mean age of 59.9 (SD16.2) years, 48% female, 50% born in Australia, 45% with high school or lower education, and 56% with low-medium OHL. A subgroup of 21 participants with a mean age of 68.1 (SD14.6) years, 40% female, 64% born in Australia, 56% with a high school or lower education, and 45% with low-medium OHL completed the interview. Three themes identified from the interviews included 1) attitudes and perceptions about oral health that highlighted a lack of agency and low prioritisation of oral health, 2) limited knowledge and education about the causes and consequences of poor oral health, including limited access to oral health education and finally 3) barriers and enablers to maintaining good oral health, with financial barriers being the main contributor to low OHL.

**Conclusions:**

Strategies aimed at redressing disparities in oral health status should include improving access to oral health information. The focus should be on the impact poor oral health has on general health with clear messages about prevention and treatment options in order to empower individuals to better manage their oral health.

**Supplementary Information:**

The online version contains supplementary material available at 10.1186/s12889-023-16381-5.

## Background

The World Health Organisation (WHO) identifies oral health as a key indicator of overall health, well-being and quality of life [1]. Poor oral health due to oral disease can result in toothache, fair/poor self-rated oral health, discomfort with appearance and food avoidance [2]. The WHO reports that the burden of oral disease is highest in socioeconomically disadvantaged groups and is projected to increase due to the growing consumption of sugar and tobacco use, particularly in developing countries [3]. Likewise in Australia, those with a lower socio-economic status (SES) are disproportionately affected by poor oral health, the incidence of which has been on an upward trajectory since 2004 [4].

Untreated, oral disease including tooth decay and periodontitis (gum) disease can lead to tooth loss. A dentition consisting of fewer than 21 teeth, is considered non-functional [5] and results in compromised chewing ability which in turn can lead to low body mass index (BMI) and undernutrition [6]. There is also now substantial evidence indicating that periodontitis increases the risk for cardiovascular disease (CVD) [7], and hypertension [8] and has a bi-directional relationship with diabetes [9]. Poor oral health also has an economic cost. In 2015 world-wide costs due to dental disease were reported to be $544 billion, ranking third behind diabetes and CVD [10]. In Australia, 61% of the cost of dental care falls to the individual, 25% of the cost is covered by the government, and 14% by health insurance funds [11].

Oral health literacy (OHL) is defined as the “degree to which individuals have the capacity to obtain, process and understand basic oral health information and services needed to make appropriate health decisions” [12]. Poor OHL has been proposed as a contributing factor in disparities in oral health outcomes [13] and as such has been shown to be associated with increased negative oral health impacts including dental emergencies, an increase in dental decay and poor periodontal health [14, 15]. Therefore, improving OHL is recognised as an important priority for promoting better oral health outcomes, particularly among those experiencing social disadvantage.

Western Sydney is an area in NSW, Australia which has a population with a highly diverse social and cultural profile and a relatively high proportion of persons in the bottom quintiles of socioeconomic status (SES) [16]. Previous research on OHL in this region has found that patients with CVD have poor knowledge about oral health and receive little information relating to oral health from cardiac health staff [17]. These findings suggest that there is a need for better integration of oral health into chronic disease management programmes such as cardiac rehabilitation. Currently, there is a lack of coordinated oral health literacy programmes for priority population subgroups in Western Sydney and very limited integration of oral health information into broader chronic disease management.

This study aims to investigate OHL among adults from disadvantaged population segments in Western Sydney in order to inform the design and development of strategies to improve oral health literacy and contribute to policy and programmes to reduce disparities in oral health status.

## Methods

### Study design

The study has a mixed methods design, with oral health literacy assessed using the short form Health Literacy in Dentistry scale (HeLD-14) [18] and insights on aspects of oral health knowledge, attitudes and perceptions gathered using semi-structured interviews. The study was approved by the Western Sydney Local Health District Human Research Ethics Committee (protocol number: 2021/ETH12053).

### Participants and recruitment

Participants were adults over the age of 35 who were receiving dental treatment at the Westmead Centre for Oral Health (WCOH). The WCOH is a public dental clinic that also provides clinical training facilities for dental students and serves individuals who have a low income. The participants were part of a larger study investigating perceptions of oral health related mobile phone applications, therefore the inclusion criteria included adults ≥ 30 years, possession of a smart phone and willingness to have an oral health application installed on their phone. The exclusion criteria were not being able to read and understand English and having cognitive impairment. Participants were recruited on site using convenience sampling at the WCOH from March 2022-September 2022 at the WCOH by a research assistant who attended the clinic on selected days. Participants who completed the OHL survey and elected to undertake the semi-structured interviews were included in a sub-sample which comprised a range of participants in terms of age, gender and culturally and linguistically diverse backgrounds.

### Data collection

The survey completed by study participants measured OHL using the HeLD-14 questionnaire [18] which was developed to estimate the capacity of an individual to obtain, process or interpret, and understand basic oral health information and services needed to make appropriate oral health-related decisions [18]. The instrument reflects the multi-dimensional nature of OHL and contains 7 domains and 14 questions. The survey was administered by tablet using an online form hosted through the research electronic data capture (REDCap) platform. Each of the 14 HeLD questions was scored using a 5-point Likert scale ranging from 0 (“Unable to do”) to 4 (“without any difficulty”) and summed to obtain an overall score. The possible range of HeLD-14 scores is from 0 (lowest oral health literacy) to 56 (highest oral health literacy). Additional questions about participant characteristics included age, gender, country of birth, education level, number of teeth and frequency of tooth brushing.

For the semi-structured interviews, a guide (Additional file 1) was developed based on a previous study that explored oral health knowledge in pregnant Australian women [19]. Broad topics covered in the interview included perceptions of current oral health status, importance of oral health compared to overall health, consequences of poor oral health, knowledge and use of preventive measures, and barriers and enablers to achieving good oral health. The interviews were conducted one-on-one by telephone, with an average duration of 30 min (ranging from 15–60 min). All the interviews were conducted by the same research assistant (AT) who had experience in qualitative research and were continued until data saturation appeared to have been reached. These were audio recorded and field notes were taken during the interview. Participants did not receive any incentives for completing the interviews. They were not able to review or edit their responses, however they were free to withdraw from the study at any point.

### Data analysis

Descriptive characteristics were analysed by calculating absolute values (n) and percentages (%). HeLD-14 scores were categorised into tertiles: ≤ 35 (low OHL), 36–46 (medium OHL) and > 46 (high OHL).

Audio recordings of interviews were transcribed, and the data imported and managed within NVivo 12 [20]. Thematic analysis was conducted following the six stages proposed by Braun and Clarke [21] which included familiarisation with the data, generation of initial codes, searching for themes, reviewing themes and sub-themes, and defining and naming themes and sub-themes [21]. A sample of transcripts were independently coded by (SK) and (AT) and the codes grouped into preliminary themes and sub-themes. These transcripts and themes were reviewed by an experienced qualitative researcher (LL). Following this review the remaining transcripts were coded by (SK) and (AT). Final codes and themes were reviewed by co-authors to ensure that the themes accurately reflected the interview data. Any points of disagreement regarding coding or thematic grouping were discussed with co-authors in order to reach consensus. This component of the study follows the “Consolidated criteria for reporting qualitative research” (COREQ) checklist for reporting qualitative research [22].

## Results

A total of 51 people were recruited, 3 participants withdrew and were excluded from the analytic sample. The HeLD-14 questionnaire was completed by 48 participants, a subgroup of 21 participants completed the interview. The mean participant age for the survey group was 59.9 (SD 16.12) years, 48% were female (Table 1), 50% were born in Australia and the remaining were represented by 16 different countries and 11 different languages. A college or university level education was attained by 35%, technical level education (TAFE) by 19%, secondary school level by 37% and primary school level education by 8% of the survey group. The mean age of interview participants was 68.1 (SD14.6) years; 43% were female and 66% were born in Australia (Table 1). A college or university level education was attained by 24%, TAFE level education by 19%, secondary school level by 42% and primary school level education by 14%. Most participants brushed twice a day in both the survey group and interview subgroup (59%, 62% respectively). The number of participants with a non -functional dentition (fewer than 20 teeth) was 46% in the survey group and 71% in the interview subgroup. The OHL scores were low-medium in 56% of the survey group, and 42% in the interview group. In the interview subgroup, the majority of participants self-reported their oral health as poor.

**Table 1:** Participant characteristics in the survey group and interview sub-group

Enrolled: 51, withdrew: 3		
**Total participants – HeLD-4 Questionnaire: 48, OHL interview: 21**		
	**Survey**	**OHL interview**
Age	60 (16.11)	68 (14.59)
Female, n (%)	23 (48%)	9 (43%)
Australian born, n (%)	24 (50%)	14 (66%)
**Educational attainment**		
College or University	17 (35%)	5 (24%)
Technical college (TAFE)	9 (19%)	4 (19%)
Secondary school	18 (38%)	9 (43%)
Primary school	4 (8%)	3 (14%)
**Number of Teeth**		
1–9 teeth	5 (11%)	4 (19%)
10–19 teeth	17 (35%)	11 (52%)
≥ 20 teeth	25 (52%)	5 (24%)
Did not specify	1 (2%)	1 (5%)
**Brushing frequency**		
Twice a day	29 (60%)	13 (62%)
Once a day	17 (35%)	6 (29%)
Less than once a day	2 (5%)	2 (9%)
**OHL Score**		
≤ 35 (Low)	4 (8%)	2 (9%)
35–46 (Medium)	23 (48%)	8 (36%)
> 46 (High)	20 (42%)	12 (55%)

### Scores in domains of oral health literacy

As shown in Table 2, the major contributing factor to low OHL included economic barriers which received a score below 2 (indicating that most were unable or found it very difficult to pay to see a dentist, or to pay for medication to manage their oral health). Other factors that included scores below 3 (indicating that participants were unable to, found it very difficult or had some difficulty performing tasks) included obtaining a second opinion about their dental health (2.33), the ability to pay for medication to manage their oral health (2.52), the ability to pay attention to their oral health (2.60), and obtain support from family or friends for dental visits (2.88 and 2.96 respectively).

**Table 2:** Participant scores for individual questions on the HeLD -14 questionnaire

Question	Domain	Score
Ability to pay attention to your dental or oral health	Receptivity	2.60
Ability to make time for things that are good for your dental or oral health?	Receptivity	3.35
Ability to read written information, for example, leaflets given to you by your dentist?	Understanding	3.56
Ability to read dental or oral health information brochures left in dental clinics and waiting rooms?	Understanding	3.49
Ability to take family or a friend with you to a dental appointment?	Utilisation	2.88
Ability to ask someone to go with you to a dental appointment?	Utilisation	2.96
Ability to pay to see a dentist?	Economic barriers	1.48
Ability to pay for medication to manage your dental or oral health?	Economic barriers	2.52
Ability to get a dentist appointment?	Access	3.79
Do you know what to do to get a dentist appointment?	Access	3.72
Ability to look for a second opinion about your dental health from a dental health professional?	Communication	2.33
Ability to use information from a dentist to make decisions about your dental health?	Communication	3.56
Ability to carry out instructions that a dentist gives you?	Utilisation	3.60
Ability to use advice from a dentist to make decisions about your dental health?	Utilisation	3.67

Scores (0 = unable, 1 = Very difficult to perform, 2 = Neutral, 3 = With a little difficulty, 4 = Without any difficulty

### Factors contributing to oral health literacy

The major themes identified from qualitative analysis of the interviews included attitudes and perceptions about oral health care, limited knowledge and education about oral health and barriers and enablers to managing oral health. These 3 themes were expanded into subthemes, as is presented in Table 3, and their relevance to the seven domains of OHL assessed by the HeLD-14 is examined below.

**Table 3:** Major themes and subthemes concerning oral health

THEME	SUB THEMES
**Attitudes and perceptions about oral health**	▪ Lack of agency/control over oral health
	▪ Low prioritisation of oral health
	▪ Misconceptions about preventive oral health practices
**Limited knowledge and education about oral health**	▪ Limited knowledge about the consequences of poor oral health
	▪ Limited knowledge about the causes of poor oral health
	▪ Limited access to oral health education
**Barriers and enablers to managing oral health**	▪ Barriers:
	- Economic barriers
	- Fear of dental treatment
	- Long waiting lists for dental care
	- Limited access to transport
	- Poor delivery of oral health information
	▪ Enablers:
	- Better information about available oral health services and
	- Better delivery of oral health information
	- Access to universal dental records
	- Access to regular check-ups
	- More timely oral health advice

### Receptivity

The scores for the domain of receptivity included 2.60 for the participants ability to pay attention to their oral health and 3.35 for the ability to make time to look after their oral health (Table 3).

The interview data suggests that the reason many participants were unable to or found it very difficult to pay attention to their oral health may be explained by their attitudes and perceptions about oral health. Several participants reported that they lacked agency over their oral health. This was reflected in either the expectation that oral health declined with age (Table 4, quote 1) or a sense of resignation about their poor oral health status in terms that nothing would prevent further deterioration or that they just wanted the teeth removed (Table 4, quotes 2–3). Additionally, although many participants believed that they had poor oral health (Table 4, quote 4), most only prioritised oral health care when problems developed (Table 4, quote 5). Furthermore, a common reflection was that they were often not motivated to perform routine oral hygiene practices due to laziness, and also because they prioritised other health issues over oral health (Table 4, quote 6–8). Previous bad dental experiences resulted in fear and avoidance of dental treatment (Table 6, quote 5). There were also misconceptions reflected in a belief that dental visits were required to address problems rather than for routine preventive care (Table 4, quote 9). One participant commented that routine dental visits were not required for young people (Table 4, quote 10).

**Table 4:** Attitudes and perceptions about oral health

**Lack of agency/control over oral health**
1. I know some people maintain good standard of teeth through to old age but I think quite a few of them have had – even younger than me – replacements and dentures (…..) I’m nearly seventy so, it would be unsurprising that my teeth were deteriorating somewhat (P13)
2. Should brush twice a day, but I think it’s too late. [Laughs] You know? I’ve only got eleven bottom teeth left (P28)
3. I brush, I use the toothpaste that you buy from the chemist. Like I try and do everything but there’s nothing that can sort of save them—now it’s just sort of trying to prevent more damage but I just want them ripped out. Just give me dentures, I don’t care (P7)
4. Well, I've never liked them. They've always been crooked. And now they're just- they just keep falling out. They’re just no good (P26)
**Low prioritisation of oral health**
5. Well, up until two years ago I suppose, or a year and a half ago, I didn’t sort of think about it too much. Occasionally I’d go to the dentist and get a filling here and there. But for some reason, in the last two years, my teeth have deteriorated pretty badly. So, you know now, it’s pretty important (P2)
6. Oh, I think it's just, I never considered looking after them- it's just laziness I guess. It's not part of my normal routine [laughs]. But it is becoming part of my normal routine. I have a lot of other health issues and I've been concentrating on them more (P8)
7. People have to realise the importance and that's what motivates you. You know? The importance. I mean, look at all the people that gave up smoking when those adds came out with those horrible things on the packets (….). When they saw those images. Yeah. I think if people knew what can happen to you, when you don’t look after your teeth. Maybe warnings, you know? (P21)
8. I tend to neglect my health a little bit. I suffer from depression and I get days when I can’t do anything (P2)
**Misconceptions about preventive oral health practices**
9. Well, you know, the bottom line is to be able to go and get these problems looked at on a regular basis (…….) I’d love to be able to – when I’ve got an abscess or a toothache or whatever – that I could just- because sometimes you have to wait quite a while and when you’ve got an abscess (P21)
10. If you’re younger you don’t need to check your teeth every year. You can check ten years or twenty years. But after fifty years of old, you need one year. Like I see my eye doctor, and my ears doctor, every one year (P5)

Another reason for reduced receptivity was limited knowledge and education, which resulted in reduced awareness of the impact of poor oral health on general health beyond the local effects upon the dentition and chewing function (Table 5, quotes 1–4). General health impacts that were reported included the impact on mental health (Table 5, quote 5), and a personal experience relating to a brain infection stemming from a tooth abscess (Table 5, quote 6). One participant reported that poor oral health was likely to impact their general health but was unaware of what these impacts might be (Table 5, quote 7). There was also limited knowledge about the causes of poor oral health. Whilst there was acknowledgement that routine cleaning was important (Table 5, quote 8), there was very limited understanding of the role of diet. Participants alluded briefly to the impact of acid (Table 5, quotes 9–10) and sugar (Table 5, quote 11) on oral health. There was broad awareness that smoking was likely to negatively impact oral health, however there were also misconceptions regarding how smoking might affect the teeth (Table 5, quote 12). Importantly, several participants did not know why their oral health had deteriorated (Table 5, quote 13).

**Table 5:** Limited knowledge and education about oral health

**Limited knowledge about the consequences of poor oral health**
1. Bad breath, cavities, gum disease, gingivitis – I think that’s what it’s called – that can come about as well. Also tooth loss, mouth ulcers (P28)
2. Like a lot of it (poor oral health) is appearance to be honest. I can’t smile at all. Like I’ll smile with my lips closed (P7)
3. I can’t eat certain foods that I used to be able to ‘cause I’m just missing basically a lot of teeth. It just takes me so long to chew food and it just takes the enjoyment out of eating (P19)
4. sometimes you have to wait quite a while and when you’ve got an abscess—I don’t know if you’re familiar with how painful they are (….) but it’s extremely, extremely painful
5. it impacts your system, impacts how you feel about yourself. If you don't feel good about yourself, you know what I mean? You won't socialise, you won't smile, all that sort of stuff, you know? Which impacts on your overall mental health in my opinion (P38)
6. I had a sister in 1957 who got an abscess on a lower jaw and she finished up with an infection in her brain and she got encephalitis (P21)
7. Well, I suppose if you let it go… You know, your whole system is going to suffer. But I don’t think mine has. So, I can’t really comment on that with any certainty (P19)
**Limited knowledge about the causes of poor oral health**
8. Neglecting the routine cleaning and that sort of thing (P1)
9. Lemon juice actually rots your teeth. It burns off the veneer (…….) and you know literally ruins your teeth (P22)
10. I don’t know, I love biccies, I love cake and I say that that might be what’s done a lot of damage (P21)
11. I had stomach surgery and it caused a lot of, like, acid. There was a lot of reflux and so, that started to eat away at my teeth …. (P7)
12. You know, I lost my teeth because I was a heavy smoker. Kind of like self-inflicted misery. It's gum disease of the upper teeth. Heavy smokers get it. But they don’t lose their bottom teeth, they lose their upper teeth. It doesn’t really affect the bottom teeth much (P28)
13. Although I’ve got a lot of problems with my teeth and you would say, well what have I done wrong for my teeth to be this way? But I’m not sure. I don’t know (P2)
**Limited oral health education**
14. Oh! He would tell me, you know, because I have teeth out and he said, “[P17’s name], you shouldn’t do this and do that”. If I went to the dentist. But I never receive anything to read (P17)
15. To tell you the truth I'm going back- every dentist I've had, I can't honestly remember any of them, giving me any literature or anything about dentistry to sort of, you know, save my teeth other than the one that told me that, um, basically I'd smoke my teeth to death (P22)

### Understanding

Many participants had little difficulty with this domain which included the ability to read written information provided by a dentist, and the ability to read general oral health information found in dental clinics. The qualitative data explored the details on preferences for receiving information. Although over 50% of participants were happy with educational material being delivered by a variety of sources including electronic or hard copy format, some formats such as text messages were reported as an acceptable format by only 3 participants. An older participant explained how written material was sometimes hard to follow if it contained too much jargon (Table 6, quote 9). Face-to-face interactions were preferred by 3 participants (Table 6, quote 15).

**Table 6:** Barriers and enablers to maintaining good oral health

**Economic barriers**
1. You asked me if I could afford to do this myself. The price I paid for that one filling is what I get for a fortnight. (…..) My pension is, $740 a fortnight, and I either pay $640 for one tooth. So, [laughs] I can't afford these things (P45)
2. In those days, only one parent worked, and we didn't have money for dental health(….). You only went to a dentist if there was a problem (P38)
3. After retiring, we can't afford the private health cover. It's too much for two adults at that age to come out of your income. And there's a lot of stuff that they don't cover anyway, you know, so you still of pocket whichever way you do it. I think there's not enough cover for sure teeth, you know (P38)?
4. I needed to get a tooth pulled out because it had an abscess and I couldn’t afford it. So, I go to the doctor and get an antibiotic to stop the swelling and get some more time until I could afford to do it (P2)
**Other barriers (fear, long waiting lists, transport, poor delivery of oral health information)**
5. I've seen her (current dentist) quite a bit. And I'm not so nervous now. But, you know, it's one of the reasons why over the years, I haven't visited the dentist unless I really had to (P8)
6. I'm 72, 2015, my wife died. That was the last time I saw the dentist. I had a problem then, I booked in and—they said, “Oh, you know, it'll be a three year wait (P22)
7. I honestly think we've got a very good health system here, but not enough in the dental. I waited now for years and I haven't heard from them (P38)
8. Yeah, I have to depend on community transport. So, I've got to book that a week in advance (P8)
9. sometimes when they send an email, they put a hell of a lot of garbage in with it and they put different, words in that- I can't remember. I'm looking for a word that fits… jargon. They put jargon in and to me, if you put jargon into me, it's straight out (P22)
10. They’re not taking them all out. I’m not sure. They haven’t really talked to me too much about what I can get as far as replacement teeth or anything like that (…..)there was no talk about what they can do for me or what I should do in the future to address the issues (P2)
**Enablers: Better information about available oral health services and support for oral health care**
11. This is a free dental health check-up. I happen to know it through Service New South Wales when I went there and they told me that there are some benefits and discounts for seniors (…….) without me being at the Service New South Wales office I wouldn't know that there is such a thing (P1)
12. They (different state) pay- I think she said it was like a $120 or something like that. Like quite a small amount and then they get something like $1800 worth of dental care and they get that every year (….) I would pay that, you know, even $200 or $300, I could afford that (P7)
13. It would be good also if the state government or even the federal would tie up with the private insurance companies to make the cost of oral health more affordable (P1)
**Enablers: Better delivery of oral health information**
14. He actually sat down and had a discussion and told me that I should do this and I should do that. And he gave me reasons why. He didn't just turn around and say, “You got to brush your teeth! He actually explained why I should brush my teeth, why I should do this and, and all that. And I listened (P22)
15. You know exactly where you stand when it’s face-to-face. (….) if you don’t understand when you’re face-to-face you can ask the questions, “Is this what you’re talking about? Is this correct?” You can’t- well, you can, but it’s a very convoluted thing with any other form (P19)
**Other enablers (universal dental records, oral health advice)**
16. if you go to a different dentist than what you went to previously, (…) they don’t have the records (….) So, you have to explain things over again and sometimes you don’t understand it yourself. So, if there was some record keeping that would be really cool (P7)
17. Well, it's the brushing method, the length of time (…).. They also told me about not rinsing off the toothpaste. And they got me using those little Christmas tree pics as well. I'm sorry I was 75 before I learned all this. It would’ve been handy to know 50 years ago (P21)

### Utilisation

The domain of utilisation is listed twice in the HeLD-14 assessment; in the first instance it relates to the availability of support for dental visits and in the second instance to the ability to act on information received.

Although the need for support to attend dental visits scored below 3 it was rarely mentioned in the interviews apart from one participant who reported that they were dependent on community transport (Table 6, quote 8). In terms of carrying out instructions and acting on advice, the interviews did reveal some areas of need. Providing the rationale for preventive practices was reported to be more helpful in achieving behaviour change (Table 6, quote 14). Oral health care instructions from oral health care providers were welcomed and would have been appreciated when the participants were younger (Table 6, quote 17). Significantly one participant noted that a lack of information from their treating dentist made it difficult to know how to manage their care in the future (Table 6, quote 10).

### Economic barriers

The domain of economic barriers was the major contributing factor to low OHL in this study... Similarly, in the interviews over 50% of participants reported that the cost of dental care limited their ability to see a dentist (Table 6, quote 1). Cost also affected the ability to seek routine care (Table 6, quote 2). One participant commented that following retirement private health insurance was unaffordable and left them out of pocket for dental expenses (Table 6, quote 3). Financial constraints also resulted in participants seeking treatment from their medical practitioner for dental problems (Table 6, quote 4).

### Access

The domain of access asks about their knowledge on how to get a dental appointment, and their knowledge on what to do to get a dental appointment. Although these items scored above 3 indicating that most participants did not have difficulties with access, there were several participants who commented that they had been unaware of the availability of the public dental services (Table 6, quote 11). Participants also commented that Government support to subsidise dental care (Table 6, quote 12), or work with insurance companies to make care more affordable (Table 6, quote 13) would improve their ability to attend dental visits. Other barriers that might impact access included transport issues (Table 6, quote 8).

### Communication

The domain of communication includes the ability to look for a second opinion and the ability to use information from a dentist to make decisions about oral health. Many participants had difficulty with the ability to look for a second opinion which likely relates to the economic barriers to accessing dental care and the dependence that these participants had on the public dental system. The interview analysis revealed that the long waiting times in the public system were a significant barrier to obtaining dental care (Table 6, quote 6). There was acknowledgement that the overall health system was quite good but that the dental system was not (Table 6, quote 7). Additionally, the lack of universal dental records made it hard to transfer to different dentists (Table 6, quote 16). Of note is that almost all participants reported that they had received very limited oral health information in the past (Table 5, quotes 14–15). Several mentioned that they had received the information very late in life and that it would have been more helpful to receive this when they were younger and that they appreciated information on brushing technique, use of interdental brushes (Table 6, quote 17).

## Discussion

This mixed-method study has provided insights into the multiple factors affecting OHL in a social disadvantaged population. This study demonstrates that while OHL may be improved by more targeted communication and education strategies, these need to be supported by service level and systemic changes to reduce barriers to preventive oral health care. Almost half the study population had low to medium OHL, and many had poor oral health as reflected in the levels of tooth loss and the proportion brushing once a day or less. Analysis of the interview data revealed 3 major themes including attitudes and perceptions about oral health, limited knowledge and education about oral health, and barriers and enablers to maintaining good oral health.

Poor understanding of the impact poor oral health could have on their general health was a key factor affecting the ability of participants to pay attention to their oral health. The impact of poor oral health, particularly gum disease on oral health is well established. Evidence from observational studies have consistently shown that periodontitis (gum disease) increases the risk for poor CVD outcomes [7], increases the risk of developing hypertension [8] and has a bidirectional relationship with type 2 diabetes mellitus (T2DM) [9]. Furthermore, there is now a growing body of evidence to suggest that tooth loss is associated with other general health conditions such as cognitive decline [23], and frailty [24]. However, despite this evidence many focused on the local effects such as pain, tooth loss, and the impact on diet and appearance. Similar findings have been reported in patients with CVD, where despite a high incidence of oral health problems many lacked knowledge about the importance of good oral health and the association between poor oral health and heart disease [17]. Furthermore, although many claimed to value oral health, they often listed it as a low priority until problems developed.

Most participants understood that oral hygiene practices such as tooth brushing, interdental cleaning were important in maintaining good oral health. However, despite established recommendations for twice daily brushing, 38% of interview participants and 40% or survey participants were only brushing once a day or less. This may be explained by the sentiment expressed by some that they did not feel there was any point in trying to look after their teeth as they were too far gone. The goal of tooth brushing and interdental cleaning is to reduce the oral bacterial load. The build-up of oral bacteria triggers a localised inflammatory response in the tissues which can escalate and contribute to the overall inflammatory risk profile for diseases such as CVD [25]. A cohort study of over 200,000 participants found that an additional brushing of the teeth per day was associated with a 9% reduction in the risk for CVD [26].

Many participants in the study had poor oral health knowledge about key preventive oral health behaviours. Sugar consumption has a significant impact on the risk for tooth decay [27], yet it was mentioned by only a few participants. Consuming adequate water to ensure hydration helps maintain saliva flow which in turn protects against both tooth decay and gum disease [28]. However only one participant noted that they should drink more water. Other factors that can affect hydration such as caffeine intake were not mentioned and even the impact of certain medications on saliva flow was poorly understood. This lack of knowledge about general preventive behaviours in a population with low-medium OHL mirrors findings from a recent study that showed that those with higher oral health literacy were more likely to engage in preventive dental care [29]. The lack of agency many participants felt in relation to their oral health may be due to this lack of oral health knowledge and is likely compounded by social disadvantage. This disadvantage is reflected in the fact that the highest level of educational attainment for 45% of the study population was at the high school level (and for 8% at the primary school level). The findings from this study are consistent with evidence that indicates that poor OHL is associated with poor oral health outcomes, poor oral health behaviours [14] and poor oral health knowledge [30].

Although the participants in this study scored well on items relating to using information from a dentist the interview data suggests that many participants received very little information on oral health, and that when information was received it was from their dentist. Preventive information delivered by oral health care practitioners has been shown to improve oral health outcomes, particularly in reducing tooth decay [31]. However, limited access to oral health care means that access to preventive information is also limited. Our findings are not dissimilar to a large qualitative study involving 6 European countries and over 140 participants that reported a lack of patient education and oral health awareness across Europe [32].

The major factor that contributed to a low OHL score were the economic barriers, and this was consistent with the interview data that highlighted financial issues as the main reason participants were not able to seek dental care. Nationally, Australian Bureau of Statistics data report that every year 2 million Australians defer visits to a dentist due to cost [33]. Internationally, in a 2014 National Health Interview survey from the United States of America the cost of treatment as a barrier to accessing care was reported by more people for dental care compared to any other type of health care [34]. The interview data identified that the cost of dental care limited not only access to see a dentist, but also the treatment options and the ability to receive routine care.

Taken together the findings from this study suggest that there is a critical need to engage with the community to improve awareness of the broader implications of poor oral health on general health and to improve the delivery of preventive information. The focus of dental care is often on treatment rather than prevention [35]. However, dental disease is preventable, therefore a stronger emphasis on preventive care will help reduce the community burden of dental disease. Delivering preventive information using multiple platforms including digital media and better utilisation of the dental team (the dentist, oral health therapists, hygienists and nurses) could improve information dissemination. Additionally, oral health education could be integrated into chronic disease management such as diabetes counselling, cardiac rehabilitation, and healthy aging programmes. Despite sharing common risk factors, oral health information is often limited or absent from these programmes and there is a growing awareness of the need for better integration of oral and general health care [36].

### Strengths and limitations

The strength of this study is the richness of the interview data from a diverse population group with a high burden of dental disease. This data provides insight into how future oral health messaging and prevention strategies could be designed for diverse and higher risk population groups.

One of the limitations is that although participants in this study were drawn from a range of cultural backgrounds, we were not able to include non-English speaking participants due to the lack of funding for translation services. Furthermore, participants were not provided with the opportunity to review or edit their comments, and this may impact the validity of the conclusions. Another limitation is the small sample size and method of participant selection using convenience sampling. These issues limit the ability to generalise the findings from this study to the broader population. Finally, findings regarding the perceived lack of control and resignation about oral health status may be skewed due to the older age of participants in this study. However, this is an important finding as any oral health information designed for older adults should address these issues. Future studies could focus on using translation services to collect information from non-English speaking participants to get a broader understanding of oral health related knowledge, attitudes and perceptions of these individuals.

## Conclusion

This mixed methods study identified that in a socially and culturally diverse population of Australian adults with a high burden of oral disease, many have low to medium oral health literacy. The major factor contributing to reduced oral health literacy was the high cost of oral health care. This was compounded by limited knowledge about the causes and consequences of poor oral health, a lack of agency and low prioritisation of oral health. To improve oral health outcomes in this community strategies are required to better empower individuals to manage their oral health, increase the prioritisation of oral health care, and overcome the barriers to accessing care.

## Supplementary Information

**Additional file 1. **Interview guide 

## Data Availability

The datasets used and/or analysed during the current study are available from the corresponding author on reasonable request.

## Abbreviations

BMI: Body mass index
CVD: Cardiovascular disease
HeLD-14: Health Literacy in Dentistry scale
OHL: Oral health literacy
REDCap: Research electronic data capture
SES: Socioeconomic status
T2DM: Type 2 Diabetes Mellitus
WHO: World Health Organisation
WCOH: Westmead centre for oral health
