# Supplementary material for: Oral health literacy, knowledge and perceptions in a socially and culturally diverse population: a mixed methods study
Source: BMC Public Health. 2023 Jul 28;23:1446. doi: 10.1186/s12889-023-16381-5 (PMC10375643; doi:10.1186/s12889-023-16381-5)
Supplement: Supplementary file 1 — Additional file 1. Interview guide [file 12889_2023_16381_MOESM1_ESM.docx]

Suppl 1: Interview Guide

| Interview prompts | |
| --- | --- |
| Oral health status (Perceptions) | *Example: Can you describe the current status of the health of your teeth and mouth.*  *Follow-up question: Do you have any concerns about your oral health?* |
| Importance of oral health compared to overall health (Attitudes and Knowledge) | *Example: Can you tell me how important you feel the health of your teeth and mouth are compared to other aspects of your health?* |
| Consequences of poor oral health (Knowledge) | *Example: What do you think are some of the problems that can be caused by poor oral health?*  *Participant with poor oral health – How do you think the poor health of your teeth and mouth affects you? Can you provide some examples?* |
| Influences upon oral health (Knowledge) | *Example: What are the major things that affect the quality of your oral health?* |
| Preventive measures (Knowledge) | *Example: What are some of the things* ***you*** *can do to improve the health of your teeth and mouth, can you provide examples?*  *Follow up question: I would like to ask specifically about how you think particular actions affect your oral health: (use prompts below as needed)*  • Brushing your teeth  • The type of toothpaste you use  • Using dental floss or interdental brushes  • The things you drink  • The kinds of food you eat  • Visiting a dental practitioner |
| Barriers and enablers to achieving good oral health (Barriers) | *Example: Can you describe some of the things that make it difficult for you to look after your teeth and mouth?*  *Follow up question: Could you tell me about any difficulties or challenges you have with any of the following aspects of oral health? (use prompts below as needed)*  • Brushing your teeth twice a day  • Using fluoride toothpaste  • Using dental floss or interdental brushes to clean between your teach every day  • Drinking tap water regularly throughout the day  • Limiting foods high in sugar, fat and salt  • Visiting a dental practitioner for a check-up at least once per year… |
| Barriers and enablers to achieving good oral health (Enablers) | *Example: What do you think would help you to carry out these oral health practices?* |
| Information and support | *Example: Can you recall receiving any information about oral health from your dentist or another source?*  *Follow-up question 1: Was that information helpful, or unhelpful? Please tell me what was helpful or unhelpful?*  *Follow-up question 2: What other information would assist you in relation to your oral health? Use prompts as needed (causes of poor oral health, advice about ways to maintain oral health, access to oral health practitioners – dentist, hygienist etc).*  *Follow-up question 3: What would be the best ways to deliver that information to you? Use prompts as needed (leaflet, internet, digital media)*  *Follow-up question 4: Are there any ways that information could be provided through your smartphone or the internet that would be helpful?* |
